# Crystal structure, Hirshfeld surface analysis and DFT studies of 2-(2,3-di­hydro-1*H*-perimidin-2-yl)phenol

**DOI:** 10.1107/S2056989020005939

**Published:** 2020-05-05

**Authors:** Ballo Daouda, Nanou Tiéba Tuo, Niameke Jean-Baptiste Kangah, Tuncer Hökelek, Charles Guillaume Kodjo, Pascal Retailleau, El Mokhtar Essassi

**Affiliations:** a Laboratoire de Chimie Organique Heterocyclique URAC 21, Pôle de Competence Pharmacochimie, Faculté des Sciences, Université Mohammed V, Rabat, Morocco; b Laboratoire de Chimie Organique et de Substances Naturelles, UFR Sciences des Structures de la Matière et Technologie, Université Félix Houphouët-Boigny, 22 BP 582 Abidjan, Côte d’Ivoire; c Laboratoire de Thermodynamique et Physicochimie du Milieu, Université Nangui, Abrogoua, UFR-SFA, 02 BP 801 Abidjan 02, Côte d’Ivoire; d Department of Physics, Hacettepe University, 06800 Beytepe, Ankara, Turkey; e Institut de Chimie des Substances Naturelles, 1 av. de la Terrasse, 91198 Gif sur Yvette, France

**Keywords:** crystal structure, perimidine, phenol, Hirshfeld surface

## Abstract

The asymmetric unit of the title compound, C_17_H_14_N_2_O, contains two independent mol­ecules each consisting of perimidine and phenol units. The tricyclic perimidine units contain naphthalene ring systems and non-planar C_4_N_2_ rings adopting envelope conformations with the C atoms of the NCN groups hinged by 44.11 (7) and 48.50 (6)° with respect to the best planes of the other five atoms. Intra­molecular O—H⋯N hydrogen bonds may help to consolidate the mol­ecular conformations. The two independent mol­ecules are linked through an N—H⋯O hydrogen bond. The Hirshfeld surface analysis of the crystal structure indicates that the most important contributions for the crystal packing are from H⋯H (52.9%) and H⋯C/C⋯H (39.5%) inter­actions. Hydrogen bonding and van der Waals inter­actions are the dominant inter­actions in the crystal packing. Density functional theory (DFT) optimized structures at the B3LYP/ 6–311 G(d,p) level are compared with the experimentally determined mol­ecular structure in the solid state. The HOMO–LUMO behaviour was elucidated to determine the energy gap.

## Abstract 2

The asymmetric unit of the title compound contains two independent mol­ecules, consisting of perimidine and phenol units, which are linked through an N—H⋯O hydrogen bond. Intra­molecular O—H⋯N hydrogen bonds are observed in both independent mol­ecules.

## Chemical context   

1-*H* Perimidines are defined as *peri*-naphtho-fused pyrimidines (Varsha *et al.*, 2010 ▸). They were first discovered in 1874 (De Aguiar, 1874 ▸) and are characterized either by a binding deficit or an excess of π binding (Woodgate *et al.*, 1987 ▸). They are used as inter­mediates in dyes, dyeing and polymerization systems (Watanab *et al.*, 1977 ▸) and have been recognized as new carbene ligands (Bazinet *et al.*, 2003 ▸), attracting great inter­est (Bu *et al.*, 2001 ▸; Starshikoy *et al.*, 1973 ▸). 1-*H* Perimidines also exhibit important biological activities (Zhou *et al.*, 2019 ▸), having the potential to act as anti-inflammatory agents (Zhang *et al.*, 2017 ▸) and inhibitors of enzymes (Alam *et al.*, 2016 ▸) and to have applications in fluorescence (Giani *et al.*, 2016 ▸), catalysis (Behbahani *et al.*, 2017 ▸), corrosion inhibition (He *et al.*, 2018 ▸) and in coordination chemistry (Booysen *et al.*, 2016 ▸; Mahapatra *et al.*, 2015 ▸).

Perimidines are obtained by the condensation of 1,8-di­aminona­phthalene with various carbonyl groups. As a contin­uation of our research into the development of new perimidine derivatives with potential pharmacological applications, we have studied the reaction of the condensation of salicyl­aldehyde and 1,8- di­aminona­phthalene in ether under agitation at room temperature to give the title compound in good yield. The title compound was obtained for the first time and characterized by single-crystal X-ray diffraction techniques as well as by Hirshfeld surface analysis. The results of the calculations by density functional theory (DFT), carried out at the B3LYP/6-311G (d,p) level, are compared with the experimentally determined mol­ecular structure in the solid state.
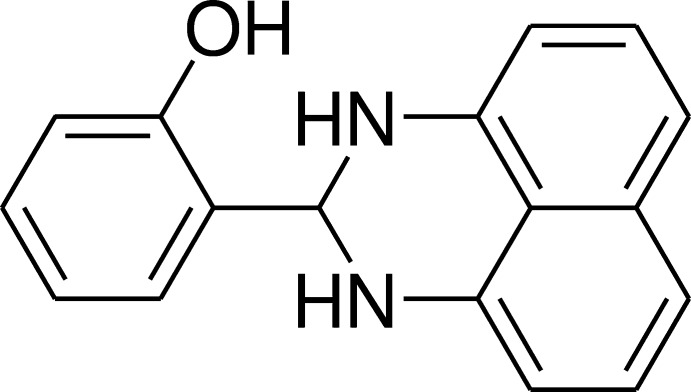


## Structural commentary   

**Figure 1:**
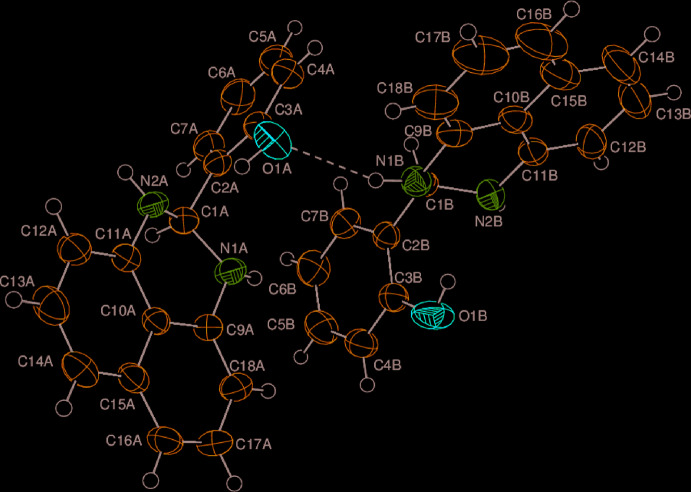
The asymmetric unit of the title compound with the atom-numbering scheme. Displacement ellipsoids are drawn at the 50% probability level.

The asymmetric unit of the title compound, **I**, contains two crystallographically independent mol­ecules each consisting of perimidine and phenol units, where the tricyclic perimidine units contain naphthalene ring systems and non-planar C_4_N_2_ rings (Fig. 1 ▸). A puckering analysis of the non-planar six-membered C_4_N_2_, *B* (N1*A*/N2*A*/C1*A*/C9*A*–C11*A*) and *B′* (N1*A*/N2*A*/C1*A*/C9*B*–C11*B*) rings gave the parameters *q*_2_ = 0.9280 (12) Å, *q*_3_ = 0.1829 (12) Å, *Q*_T_ = 0.9459 (13) Å, θ_2_ = 75.85 (15)° and φ _2_= 134.47 (18)° for *B* and *q*_2_ = 0.5320 (11) Å, *q*_3_ = 0.3791 (11) Å, *Q*_T_ = 0.6533 (14) Å, θ_2_ = 54.33 (12)° and φ _2_= −5.47 (13)° for *B′*; both rings adopt envelope conformations, where atoms C1*A* and C1*B* are at the flap positions and at distances of 0.6044 (12) and −0.6590 (13) Å, respectively, from the best planes through the other five atoms. The C_4_N_2_ rings may alternatively be described as being hinged about the N⋯N vectors with the N1*A*/C1*A*/N2*A* and N1*B*/C1*B*/N2*B* planes being inclined by 44.11 (7) and 48.50 (6)°, respectively, to the best planes through the other five atoms (N1*A*/N2*A*/C9*A*–C11*A*) and (N1*B*/N2*B*/C9*B*–C11*B*). Rings *A* (C2*A*–C7*A*), *C* (C10*A*–C15*A*), *D* (C9*A*/C10*A*/C15*A*–C18*A*) and *A′* (C2*B*–C7*B*), *C′* (C10*B*–C15*B*), *D′* (C9*B*/C10*B*/C15*B*–C18*B*) are oriented at dihedral angles of *A*/*C* = 76.78 (4), *A*/*D* = 78.49 (4), *C*/*D* = 2.09 (3)° and *A′*/*C′* = 88.43 (3), *A′*/*D′* = 88.31 (3), *C′*/*D′* = 3.26 (4)°. Intra­molecular O—H⋯N hydrogen bonds (Table 1 ▸) may be effective in consolidating the conformations of the two independent mol­ecules.

## Supra­molecular features   

In the crystal, the two mol­ecules in the asymmetric unit are linked through an N—H⋯O hydrogen bond (Table 1 ▸, Fig. 1 ▸).

## Hirshfeld surface analysis   

**Figure 2:**
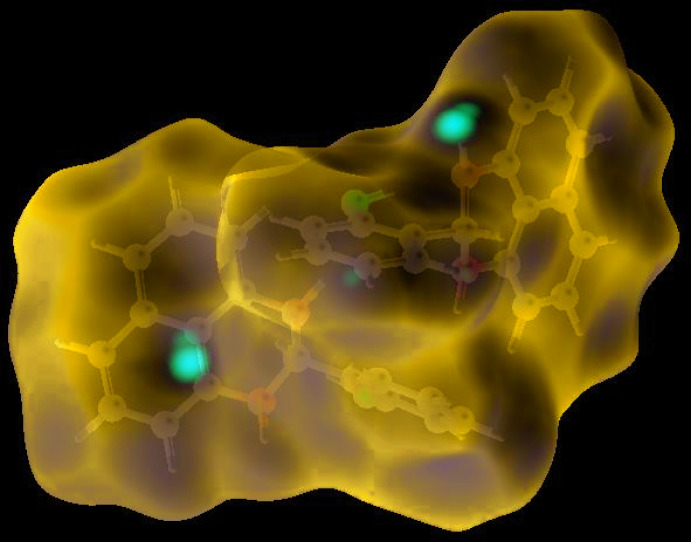
View of the three-dimensional Hirshfeld surface of the title compound plotted over *d*_norm_ in the range −0.1813 to 1.6330 a.u.

In order to visualize the inter­molecular inter­actions in the crystal of the title compound, a Hirshfeld surface (HS) analysis (Hirshfeld, 1977 ▸; Spackman & Jayatilaka, 2009 ▸) was carried out by using *Crystal Explorer 17.5* (Turner *et al.*, 2017 ▸). In the HS plotted over *d*_norm_ (Fig. 2 ▸), the white surface indicates contacts with distances equal to the sum of van der Waals radii, and the red and blue colours indicate distances shorter (in close contact) or longer (distinct contact) than the van der Waals radii, respectively (Venkatesan *et al.*, 2016 ▸). The bright-red spots indicate their roles as the respective donors and/or acceptors.

**Figure 3:**
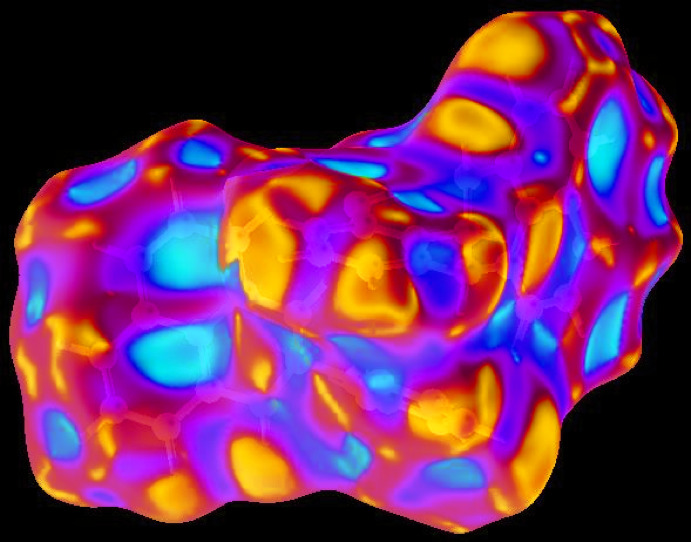
Hirshfeld surface of the title compound plotted over shape-index.

**Figure 4:**
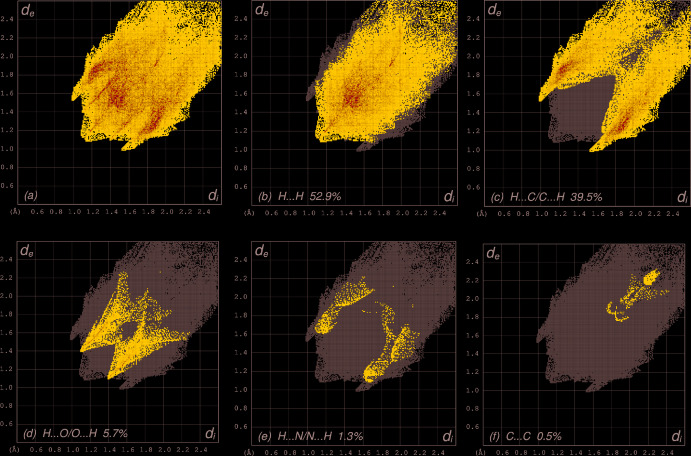
The full two-dimensional fingerprint plots for the title compound, showing (*a*) all inter­actions, and delineated into (*b*) H⋯H, (*c*) H⋯C/C⋯H, (*d*) H⋯O/O⋯H, (*e*) H⋯N/N⋯H and (*f*) O⋯C/C⋯O inter­actions. The *d*_i_ and *d*_e_ values are the closest inter­nal and external distances (in Å) from given points on the Hirshfeld surface contacts.

The shape-index of the HS is a tool to visualize the π–π stacking by the presence of adjacent red and blue triangles; if there are no adjacent red and/or blue triangles, then there are no π–π inter­actions. Fig. 3 ▸ clearly suggests that there are no π–π inter­actions in **I**. The overall two-dimensional fingerprint plot (McKinnon *et al.*, 2007 ▸) is shown in Fig. 4 ▸ *a*, and those delineated into H⋯H, H⋯C/C⋯H, H⋯O/O⋯H, H⋯N/N⋯H and C⋯C contacts are illustrated in Fig. 4 ▸ *b*–*f*, respectively, together with their relative contributions to the Hirshfeld surface. The most important inter­action is H⋯H, contributing 52.9% to the overall crystal packing, which is reflected in Fig. 4 ▸ *b* as widely scattered points of high density due to the large hydrogen content of the mol­ecule, with the tip at *d*_e_ = *d*_i_ = 1.10 Å. The pair of characteristic wings in the fingerprint plot delineated into H⋯C/C⋯H contacts, Fig. 4 ▸ *c*, (39.5% contribution to the HS) have the tips at *d*_e_ + *d*_i_ = 2.50 Å. The scattered points in the pair of spikes in the fingerprint plot delineated into H⋯O/O⋯H (Fig. 4 ▸ *d*, 5.7% contribution) have a symmetrical distribution with the tips at *d*_e_ + *d*_i_ = 2.49 Å. The H⋯N/N⋯H contacts (Fig. 4 ▸ *e*, 1.3% contribution) have a distribution of points with the tips at *d*_e_ + *d*_i_ = 2.72 Å. Finally, the C⋯C inter­actions (0.5% contribution to the overall crystal packing) are reflected in Fig. 4 ▸ *f* as low density wings with the tips at *d*_e_ + *d*_i_ = 3.60 Å.

**Figure 5:**
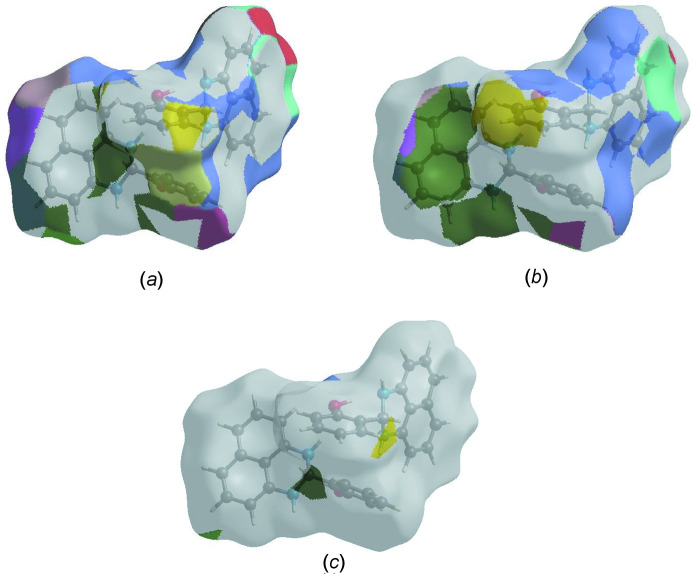
The Hirshfeld surface representations with the function *d*_norm_ plotted onto the surface for (*a*) H⋯H, (*b*) H⋯C/C⋯H and (*c*) H⋯O/O⋯H inter­actions.

The Hirshfeld surface representations with the function *d*_norm_ plotted onto the surface are shown for the H⋯H, H⋯C/C⋯H and H⋯O/O⋯H inter­actions in Fig. 5 ▸ *a*–*c*, respectively.

The Hirshfeld surface analysis confirms the importance of H-atom contacts in establishing the packing. The large number of H⋯H and H⋯C/C⋯H inter­actions suggest that van der Waals inter­actions and hydrogen bonding play the major roles in the crystal packing (Hathwar *et al.*, 2015 ▸).

## DFT calculations   

**Figure 6:**
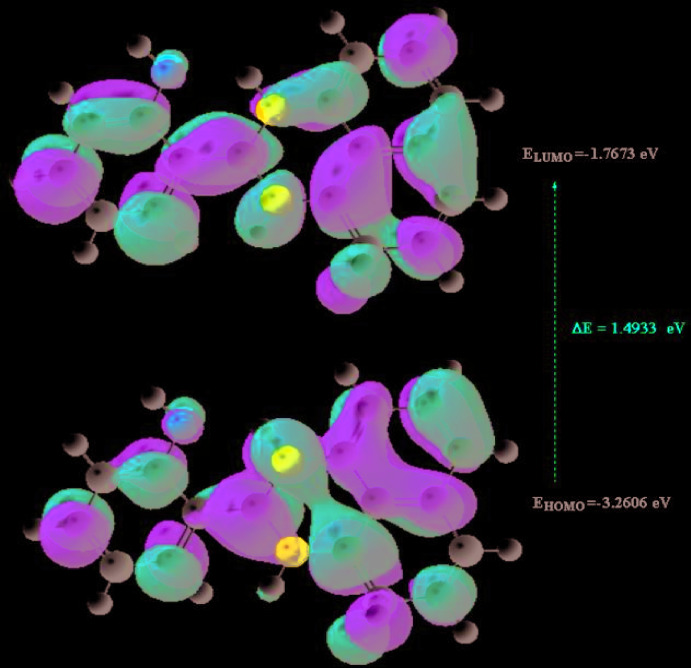
The energy band gap of the title compound.

The optimized structure of the title compound, **I**, in the gas phase was generated theoretically *via* density functional theory (DFT) using standard B3LYP functional and 6–311 G(d,p) basis-set calculations (Becke, 1993 ▸) as implemented in GAUSSIAN 09 (Frisch *et al.*, 2009 ▸). The theoretical and experimental results were in good agreement (Table 2 ▸). The highest-occupied mol­ecular orbital (HOMO), acting as an electron donor, and the lowest-unoccupied mol­ecular orbital (LUMO), acting as an electron acceptor, are very important parameters for quantum chemistry. When the energy gap is small, the mol­ecule is highly polarizable and has high chemical reactivity. The DFT calculations provide some important information on the reactivity and site selectivity of the mol­ecular framework. *E*_HOMO_ and *E*_LUMO_, which clarify the inevitable charge-exchange collaboration inside the studied material, electronegativity (χ), hardness (η), potential (μ), electrophilicity (ω) and softness (*σ*) are recorded in Table 3 ▸. The significance of η and *σ* is for the evaluation of both the reactivity and stability. The electron transition from the HOMO to the LUMO energy level is shown in Fig. 6 ▸. The HOMO and LUMO are localized in the plane extending from the whole 2-(2,3-di­hydro-1*H*-perimidin-2-yl)phenol ring. The energy band gap [Δ*E* = *E*_LUMO_ - *E*_HOMO_] of the mol­ecule is 1.4933 eV, the frontier mol­ecular orbital energies *E*_HOMO_ and *E*_LUMO_ being −3.2606 and −1.7673 eV, respectively.

## Database survey   

Similar perimidine derivatives have also been reported in which the groups at position 2 are almost coplanar with the perimidic nucleus. Examples related to the title compound, **I**, are **II** (Ghorbani, 2012 ▸), **III** (Fun *et al.*, 2011 ▸), **IV** (Maloney *et al.*, 2013 ▸), **V** (Cucciolito *et al.*, 2013 ▸) and **VI** (Manimekalai *et al.*, 2014 ▸), where **III** and **V** are most closely related while **II**, **IV** and **VI** are more distant relatives.
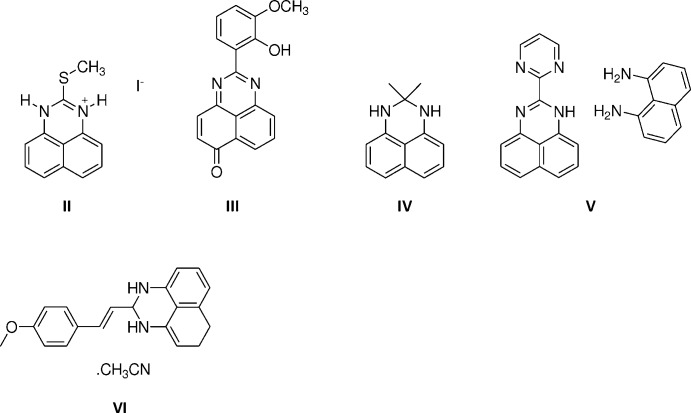


## Synthesis and crystallization   

0.35 mol (1.48 g) of 1,8-di­aminona­phthalene and 18.8 mmol (2 ml) of salicyl­aldehyde were introduced into a 250 ml flask and 30 ml of ether were added thereto. The mixture was stirred magnetically for 3 days. The grey precipitate that formed was recovered by filtration, washed with ether, rinsed with ethanol and dried under Büchner. The resulting brownish powder was recrystallized several times from ethanol to obtain colourless 2-(2,3-di­hydro-1*H*-perimidin-2-yl)phenol product (*R*_f_ = 0.70 in hexa­ne/ethyl acetate (1:0.5), yield: 97% A significant qu­antity of the colourless monocrystalline product was obtained by the slow evaporation of the solvent after 15 days.

## Refinement   

Crystal data, data collection and structure refinement details are summarized in are summarized in Table 4 ▸. The H atoms of OH and NH groups were located in difference-Fourier maps and refined freely. The C-bound H atoms were positioned geometrically, with C—H = 0.93 Å (for aromatic H atoms) and 0.98 Å (for methine H atom) and constrained to ride on their parent atoms, with *U*_iso_(H) = 1.2*U*_eq_(C).

## Supplementary Material

Crystal structure: contains datablock(s) I, global. DOI: 10.1107/S2056989020005939/lh5957sup1.cif

Structure factors: contains datablock(s) I. DOI: 10.1107/S2056989020005939/lh5957Isup2.hkl

Supporting information file. DOI: 10.1107/S2056989020005939/lh5957Isup3.cdx

Supporting information file. DOI: 10.1107/S2056989020005939/lh5957Isup4.cml

CCDC reference: 1976884

Additional supporting information:  crystallographic information; 3D view; checkCIF report

## Figures and Tables

**Table 1:** Hydrogen-bond geometry (Å, °)

*D*—H⋯*A*	*D*—H	H⋯*A*	*D*⋯*A*	*D*—H⋯*A*
O1*A*—H1*OA*⋯N1*A*	0.86 (2)	2.66 (2)	3.1072 (16)	113.8 (17)
O1*A*—H1*OA*⋯N2*A*	0.86 (2)	2.03 (2)	2.7763 (16)	144.6 (19)
O1*B*—H1*OB*⋯N1*B*	0.84 (2)	2.20 (3)	2.8835 (16)	138 (2)
O1*B*—H1*OB*⋯N2*B*	0.84 (2)	2.47 (2)	3.0196 (16)	123 (2)
N1*B*—H1*NB*⋯O1*A*	0.865 (17)	2.331 (17)	3.1608 (18)	160.8 (14)

**Table 2:** Comparison of selected X-ray and DFT geometrical parameters (Å, °)

Bonds/angles	X-ray	B3LYP/6–311G(d,p)
C1*A*—N1*A*	1.4597 (17)	1.40941
C1*A*—N2*A*	1.4646 (19)	1.35557
C1*A*—C2*A*	1.5079 (17)	1.43731
C1*A*—H1*A*	0.9800	1.03211
N1*A*—C9*A*	1.3944 (17)	1.42420
N1*A*—H1*N*1	0.873 (19)	1.00630
O1*A*—C3*A*	1.3693 (18)	1.40953
O1*A*—H1O*A*	0.86 (2)	0.97032
C2*A*—C7*A*	1.388 (2)	1.42763
C2*A*—C3*A*	1.3923 (19)	1.42630
N2*A*—C11*A*	1.4081 (17)	1.36897
		
N1*A*—C1*A*—N2*A*	106.61 (11)	115.07
N1*A*—C1*A*—C2*A*	110.09 (11)	125.03
N2*A*—C1*A*—C2*A*	109.23 (11)	109.89
N1*A*—C1*A*—H1*A*	110.3	110.17
N2*A*—C1*A*—H1*A*	110.3	110.03
C2*A*—C1*A*—H1*A*	110.3	110.08
C9*A*—N1*A*—C1*A*	117.08 (11)	117.82
C9*A*—N1*A*—H1*N*1	115.0 (12)	114.98
C3*A*—O1*A*—H1*OA*	106.1 (14)	107.84

**Table 3:** Calculated energies

Mol­ecular Energy (a.u.) (eV)	Compound **I**
Total Energy *TE* (eV)	−22880.3725
*E*_HOMO_ (eV)	−3.2606
*E*_LUMO_ (eV)	−1.7673
Gap, *ΔE* (eV)	1.4933
Dipole moment, *μ* (Debye)	3.3491
Ionization potential, *I* (eV)	3.2606
Electron affinity, *A*	1.7673
Electronegativity, *χ*	2.5139
Hardness, *η*	0.7466
Electrophilicity index, *ω*	4.2322
Softness, *σ*	1.3393
Fraction of electron transferred, *ΔN*	3.0042

**Table 4:** Experimental details

Crystal data	
Chemical formula	C_17_H_14_N_2_O
*M*_r_	262.30
Crystal system, space group	Monoclinic, *P*2_1_/*c*
Temperature (K)	293
*a*, *b*, *c* (Å)	9.0710 (4), 12.0526 (7), 24.6120 (11)
β (°)	95.999 (4)
*V* (Å^3^)	2676.1 (2)
*Z*	8
Radiation type	Mo *K*α
μ (mm^−1^)	0.08
Crystal size (mm)	0.60 × 0.35 × 0.05

Data collection	
Diffractometer	Rigaku XtaLAB PRO
Absorption correction	Multi-scan (*CrysAlis PRO*; Rigaku OD, 2018 ▸)
*T*_min_, *T*_max_	0.212, 1.000
No. of measured, independent and observed [*I* > 2σ(*I*)] reflections	29344, 6395, 4554
*R*_int_	0.042
(sin θ/λ)_max_ (Å^−1^)	0.690

Refinement	
*R*[*F* ^2^ > 2σ(*F* ^2^)], *wR*(*F* ^2^), *S*	0.046, 0.120, 1.03
No. of reflections	6395
No. of parameters	379
H-atom treatment	H atoms treated by a mixture of independent and constrained refinement
Δρ_max_, Δρ_min_ (e Å^−3^)	0.19, −0.21

Computer programs: *CrysAlis PRO* (Rigaku OD, 2018 ▸), *SHELXT2014/5* (Sheldrick, 2015*a* ▸), *SHELXL2018/3* (Sheldrick, 2015*b* ▸) and *ORTEP-3 for Windows* (Farrugia, 2012 ▸).

## supplementary crystallographic information

### Crystal data

**Table:**

C_17_H_14_N_2_O	*F*(000) = 1104
*M**_r_* = 262.30	*D*_x_ = 1.302 Mg m^−^^3^
Monoclinic, *P*2_1_/*c*	Mo *K*α radiation, λ = 0.71073 Å
*a* = 9.0710 (4) Å	Cell parameters from 8291 reflections
*b* = 12.0526 (7) Å	θ = 2.8–29.0°
*c* = 24.6120 (11) Å	µ = 0.08 mm^−^^1^
β = 95.999 (4)°	*T* = 293 K
*V* = 2676.1 (2) Å^3^	Plate, colourless
*Z* = 8	0.60 × 0.35 × 0.05 mm

### Data collection

**Table:**

Rigaku XtaLAB PRO diffractometer	6395 independent reflections
Radiation source: micro-focus sealed X-ray tube, Rigaku micromax 003	4554 reflections with *I* > 2σ(*I*)
Rigaku Integrated Confocal MaxFlux double bounce multi-layer mirror optics monochromator	*R*_int_ = 0.042
Detector resolution: 5.811 pixels mm^-1^	θ_max_ = 29.4°, θ_min_ = 2.7°
ω scans	*h* = −12→11
Absorption correction: multi-scan (CrysAlisPro; Rigaku OD, 2018)	*k* = −15→16
*T*_min_ = 0.212, *T*_max_ = 1.000	*l* = −33→32
29344 measured reflections	

### Refinement

**Table:**

Refinement on *F*^2^	Primary atom site location: other
Least-squares matrix: full	Secondary atom site location: difference Fourier map
*R*[*F*^2^ > 2σ(*F*^2^)] = 0.046	Hydrogen site location: mixed
*wR*(*F*^2^) = 0.120	H atoms treated by a mixture of independent and constrained refinement
*S* = 1.03	*w* = 1/[σ^2^(*F*_o_^2^) + (0.0558*P*)^2^ + 0.390*P*] where *P* = (*F*_o_^2^ + 2*F*_c_^2^)/3
6395 reflections	(Δ/σ)_max_ = 0.001
379 parameters	Δρ_max_ = 0.19 e Å^−^^3^
0 restraints	Δρ_min_ = −0.21 e Å^−^^3^

### Special details

**Table:**

Geometry. All esds (except the esd in the dihedral angle between two l.s. planes) are estimated using the full covariance matrix. The cell esds are taken into account individually in the estimation of esds in distances, angles and torsion angles; correlations between esds in cell parameters are only used when they are defined by crystal symmetry. An approximate (isotropic) treatment of cell esds is used for estimating esds involving l.s. planes.

### Fractional atomic coordinates and isotropic or equivalent isotropic displacement parameters (Å^2^)

**Table:**

	*x*	*y*	*z*	*U*_iso_*/*U*_eq_	
C1A	0.56889 (14)	0.53907 (11)	0.62210 (5)	0.0396 (3)	
H1A	0.638920	0.526271	0.595013	0.047*	
N1A	0.41985 (13)	0.50532 (11)	0.60023 (5)	0.0437 (3)	
H1N1	0.4106 (19)	0.4334 (16)	0.5970 (7)	0.066*	
O1A	0.43505 (13)	0.56864 (10)	0.72308 (4)	0.0584 (3)	
H1OA	0.438 (2)	0.6117 (18)	0.6953 (9)	0.088*	
C2A	0.61653 (14)	0.47595 (12)	0.67387 (5)	0.0405 (3)	
N2A	0.56102 (14)	0.65756 (10)	0.63467 (4)	0.0435 (3)	
H1NA	0.646 (2)	0.6821 (15)	0.6495 (7)	0.065*	
O1B	0.04282 (12)	0.30406 (13)	0.63319 (5)	0.0708 (4)	
H1OB	0.043 (3)	0.318 (2)	0.6666 (10)	0.106*	
N1B	0.20034 (13)	0.37845 (10)	0.73446 (4)	0.0411 (3)	
H1NB	0.2567 (17)	0.4294 (14)	0.7230 (6)	0.049*	
C1B	0.25725 (13)	0.26647 (11)	0.72676 (5)	0.0375 (3)	
H1B	0.347428	0.254276	0.751615	0.045*	
C3A	0.54903 (15)	0.49467 (12)	0.72131 (5)	0.0443 (3)	
C2B	0.28946 (14)	0.24906 (11)	0.66876 (5)	0.0369 (3)	
N2B	0.14081 (13)	0.19020 (11)	0.74018 (5)	0.0419 (3)	
H2NB	0.1541 (17)	0.1222 (14)	0.7310 (6)	0.050*	
C4A	0.59490 (18)	0.43710 (15)	0.76890 (6)	0.0582 (4)	
H4A	0.550760	0.450973	0.800663	0.070*	
C3B	0.17999 (14)	0.26533 (12)	0.62532 (5)	0.0433 (3)	
C5A	0.70642 (19)	0.35908 (16)	0.76890 (7)	0.0661 (5)	
H5A	0.736838	0.320194	0.800742	0.079*	
C4B	0.20960 (17)	0.24421 (13)	0.57226 (6)	0.0503 (4)	
H4B	0.135102	0.252304	0.543578	0.060*	
C6A	0.77248 (17)	0.33852 (16)	0.72245 (8)	0.0645 (5)	
H6A	0.846957	0.285454	0.722639	0.077*	
C5B	0.34887 (19)	0.21132 (14)	0.56196 (6)	0.0568 (4)	
H5B	0.368794	0.198052	0.526223	0.068*	
C7A	0.72813 (15)	0.39693 (14)	0.67520 (6)	0.0519 (4)	
H7A	0.773844	0.383022	0.643784	0.062*	
C6B	0.45901 (18)	0.19793 (14)	0.60424 (7)	0.0582 (4)	
H6B	0.553804	0.177175	0.597066	0.070*	
C9A	0.35196 (14)	0.56636 (11)	0.55642 (5)	0.0378 (3)	
C7B	0.42863 (15)	0.21533 (12)	0.65727 (6)	0.0456 (3)	
H7B	0.502848	0.204217	0.685787	0.055*	
C10A	0.38759 (13)	0.68071 (11)	0.55413 (5)	0.0348 (3)	
C9B	0.15927 (15)	0.40010 (13)	0.78695 (5)	0.0444 (3)	
C11A	0.49532 (14)	0.72763 (11)	0.59321 (5)	0.0379 (3)	
C10B	0.10259 (14)	0.30998 (13)	0.81524 (5)	0.0455 (3)	
C12A	0.52565 (18)	0.83872 (12)	0.59224 (6)	0.0494 (4)	
H12A	0.597660	0.868995	0.617626	0.059*	
C11B	0.09143 (14)	0.20261 (12)	0.79161 (5)	0.0422 (3)	
C13A	0.44833 (19)	0.90655 (13)	0.55310 (6)	0.0559 (4)	
H13A	0.468574	0.982195	0.553059	0.067*	
C12B	0.02694 (16)	0.11725 (16)	0.81762 (6)	0.0578 (4)	
H12B	0.018478	0.047355	0.801591	0.069*	
C14A	0.34396 (17)	0.86426 (13)	0.51502 (6)	0.0509 (4)	
H14A	0.293631	0.911237	0.489454	0.061*	
C13B	−0.0259 (2)	0.1357 (2)	0.86819 (8)	0.0763 (6)	
H13B	−0.069782	0.077655	0.885520	0.092*	
C15A	0.31142 (14)	0.75013 (12)	0.51393 (5)	0.0407 (3)	
C14B	−0.0142 (2)	0.2368 (2)	0.89238 (8)	0.0829 (7)	
H14B	−0.048187	0.246358	0.926437	0.099*	
C16A	0.20329 (16)	0.70134 (14)	0.47590 (5)	0.0508 (4)	
H16A	0.152944	0.744820	0.448781	0.061*	
C15B	0.04892 (18)	0.32860 (18)	0.86688 (6)	0.0644 (5)	
C17A	0.17231 (16)	0.59168 (15)	0.47860 (6)	0.0552 (4)	
H17A	0.100450	0.561244	0.453204	0.066*	
C16B	0.0568 (2)	0.4373 (2)	0.88808 (8)	0.0871 (7)	
H16B	0.023178	0.451434	0.921832	0.105*	
C18A	0.24558 (16)	0.52286 (13)	0.51861 (6)	0.0505 (4)	
H18A	0.222179	0.447803	0.519553	0.061*	
C18B	0.1656 (2)	0.50467 (16)	0.80913 (7)	0.0639 (4)	
H18B	0.204313	0.563464	0.790689	0.077*	
C17B	0.1125 (3)	0.5215 (2)	0.86015 (8)	0.0844 (6)	
H17B	0.115746	0.592467	0.875106	0.101*	

### Atomic displacement parameters (Å^2^)

**Table:**

	*U*^11^	*U*^22^	*U*^33^	*U*^12^	*U*^13^	*U*^23^
C1A	0.0369 (6)	0.0440 (8)	0.0368 (6)	−0.0004 (6)	−0.0008 (5)	0.0035 (5)
N1A	0.0475 (6)	0.0373 (6)	0.0431 (6)	−0.0065 (5)	−0.0108 (5)	0.0045 (5)
O1A	0.0697 (7)	0.0588 (7)	0.0487 (6)	0.0109 (6)	0.0153 (5)	0.0095 (5)
C2A	0.0347 (6)	0.0438 (8)	0.0409 (7)	−0.0056 (6)	−0.0055 (5)	0.0066 (6)
N2A	0.0470 (6)	0.0416 (7)	0.0385 (6)	−0.0103 (5)	−0.0113 (5)	0.0042 (5)
O1B	0.0442 (6)	0.1251 (12)	0.0413 (6)	0.0251 (6)	−0.0033 (5)	−0.0002 (6)
N1B	0.0451 (6)	0.0392 (7)	0.0390 (6)	−0.0011 (5)	0.0046 (5)	−0.0005 (5)
C1B	0.0320 (6)	0.0436 (8)	0.0360 (6)	0.0035 (5)	−0.0009 (5)	0.0022 (5)
C3A	0.0443 (7)	0.0458 (8)	0.0414 (7)	−0.0062 (6)	−0.0017 (6)	0.0052 (6)
C2B	0.0372 (6)	0.0352 (7)	0.0383 (6)	−0.0002 (5)	0.0039 (5)	0.0013 (5)
N2B	0.0443 (6)	0.0397 (7)	0.0424 (6)	−0.0002 (5)	0.0079 (5)	0.0026 (5)
C4A	0.0635 (10)	0.0667 (11)	0.0422 (8)	−0.0147 (8)	−0.0045 (7)	0.0124 (7)
C3B	0.0396 (7)	0.0510 (9)	0.0392 (7)	0.0017 (6)	0.0038 (5)	0.0024 (6)
C5A	0.0567 (9)	0.0738 (12)	0.0624 (10)	−0.0109 (9)	−0.0196 (8)	0.0315 (9)
C4B	0.0583 (9)	0.0533 (9)	0.0386 (7)	−0.0017 (7)	0.0010 (6)	−0.0004 (6)
C6A	0.0413 (8)	0.0676 (11)	0.0809 (12)	0.0057 (8)	−0.0115 (8)	0.0250 (9)
C5B	0.0715 (10)	0.0573 (10)	0.0441 (8)	0.0027 (8)	0.0179 (7)	−0.0076 (7)
C7A	0.0364 (7)	0.0584 (10)	0.0594 (9)	0.0023 (7)	−0.0020 (6)	0.0109 (7)
C6B	0.0520 (9)	0.0632 (11)	0.0622 (9)	0.0089 (8)	0.0195 (7)	−0.0082 (8)
C9A	0.0369 (6)	0.0431 (8)	0.0325 (6)	−0.0025 (6)	−0.0001 (5)	0.0012 (5)
C7B	0.0396 (7)	0.0461 (8)	0.0511 (8)	0.0034 (6)	0.0045 (6)	−0.0031 (6)
C10A	0.0343 (6)	0.0415 (7)	0.0288 (6)	0.0002 (5)	0.0048 (5)	0.0022 (5)
C9B	0.0421 (7)	0.0532 (9)	0.0364 (7)	0.0082 (6)	−0.0033 (5)	−0.0045 (6)
C11A	0.0416 (7)	0.0405 (8)	0.0316 (6)	−0.0032 (6)	0.0032 (5)	0.0015 (5)
C10B	0.0359 (7)	0.0647 (10)	0.0349 (6)	0.0115 (6)	−0.0008 (5)	0.0031 (6)
C12A	0.0647 (9)	0.0424 (8)	0.0401 (7)	−0.0106 (7)	0.0007 (6)	−0.0014 (6)
C11B	0.0313 (6)	0.0564 (9)	0.0381 (7)	0.0073 (6)	−0.0007 (5)	0.0114 (6)
C13A	0.0798 (11)	0.0378 (8)	0.0506 (8)	−0.0022 (8)	0.0099 (8)	0.0058 (6)
C12B	0.0459 (8)	0.0708 (11)	0.0566 (9)	0.0013 (8)	0.0049 (7)	0.0234 (8)
C14A	0.0613 (9)	0.0492 (9)	0.0427 (8)	0.0103 (7)	0.0082 (7)	0.0139 (6)
C13B	0.0614 (11)	0.1090 (18)	0.0607 (11)	0.0030 (11)	0.0162 (8)	0.0329 (11)
C15A	0.0390 (7)	0.0512 (9)	0.0327 (6)	0.0044 (6)	0.0072 (5)	0.0074 (6)
C14B	0.0694 (12)	0.137 (2)	0.0460 (9)	0.0154 (13)	0.0225 (8)	0.0196 (12)
C16A	0.0443 (7)	0.0714 (11)	0.0352 (7)	0.0037 (7)	−0.0030 (6)	0.0124 (7)
C15B	0.0549 (9)	0.0997 (15)	0.0388 (8)	0.0167 (9)	0.0057 (7)	−0.0008 (8)
C17A	0.0482 (8)	0.0757 (12)	0.0383 (7)	−0.0105 (8)	−0.0119 (6)	0.0019 (7)
C16B	0.0949 (15)	0.122 (2)	0.0457 (9)	0.0253 (14)	0.0137 (9)	−0.0216 (11)
C18A	0.0539 (8)	0.0520 (9)	0.0429 (7)	−0.0127 (7)	−0.0078 (6)	−0.0002 (6)
C18B	0.0766 (11)	0.0603 (11)	0.0528 (9)	0.0090 (9)	−0.0019 (8)	−0.0138 (8)
C17B	0.1031 (16)	0.0873 (16)	0.0611 (11)	0.0239 (13)	0.0011 (11)	−0.0309 (11)

### Geometric parameters (Å, º)

**Table:**

C1A—N1A	1.4597 (17)	C6B—C7B	1.378 (2)
C1A—N2A	1.4646 (19)	C6B—H6B	0.9300
C1A—C2A	1.5079 (17)	C9A—C18A	1.3728 (18)
C1A—H1A	0.9800	C9A—C10A	1.4181 (19)
N1A—C9A	1.3944 (17)	C7B—H7B	0.9300
N1A—H1N1	0.873 (19)	C10A—C11A	1.4154 (17)
O1A—C3A	1.3693 (18)	C10A—C15A	1.4189 (18)
O1A—H1OA	0.86 (2)	C9B—C18B	1.372 (2)
C2A—C7A	1.388 (2)	C9B—C10B	1.416 (2)
C2A—C3A	1.3923 (19)	C11A—C12A	1.368 (2)
N2A—C11A	1.4081 (17)	C10B—C11B	1.418 (2)
N2A—H1NA	0.870 (19)	C10B—C15B	1.426 (2)
O1B—C3B	1.3616 (17)	C12A—C13A	1.395 (2)
O1B—H1OB	0.84 (2)	C12A—H12A	0.9300
N1B—C9B	1.4059 (17)	C11B—C12B	1.374 (2)
N1B—C1B	1.4644 (18)	C13A—C14A	1.360 (2)
N1B—H1NB	0.865 (17)	C13A—H13A	0.9300
C1B—N2B	1.4638 (17)	C12B—C13B	1.397 (3)
C1B—C2B	1.5013 (17)	C12B—H12B	0.9300
C1B—H1B	0.9800	C14A—C15A	1.406 (2)
C3A—C4A	1.387 (2)	C14A—H14A	0.9300
C2B—C7B	1.3833 (18)	C13B—C14B	1.356 (3)
C2B—C3B	1.3949 (18)	C13B—H13B	0.9300
N2B—C11B	1.3942 (17)	C15A—C16A	1.412 (2)
N2B—H2NB	0.862 (17)	C14B—C15B	1.421 (3)
C4A—C5A	1.381 (3)	C14B—H14B	0.9300
C4A—H4A	0.9300	C16A—C17A	1.354 (2)
C3B—C4B	1.3840 (19)	C16A—H16A	0.9300
C5A—C6A	1.368 (3)	C15B—C16B	1.410 (3)
C5A—H5A	0.9300	C17A—C18A	1.401 (2)
C4B—C5B	1.373 (2)	C17A—H17A	0.9300
C4B—H4B	0.9300	C16B—C17B	1.353 (3)
C6A—C7A	1.383 (2)	C16B—H16B	0.9300
C6A—H6A	0.9300	C18A—H18A	0.9300
C5B—C6B	1.375 (2)	C18B—C17B	1.406 (3)
C5B—H5B	0.9300	C18B—H18B	0.9300
C7A—H7A	0.9300	C17B—H17B	0.9300
			
N1A—C1A—N2A	106.61 (11)	N1A—C9A—C10A	117.35 (11)
N1A—C1A—C2A	110.09 (11)	C6B—C7B—C2B	121.03 (13)
N2A—C1A—C2A	109.23 (11)	C6B—C7B—H7B	119.5
N1A—C1A—H1A	110.3	C2B—C7B—H7B	119.5
N2A—C1A—H1A	110.3	C11A—C10A—C9A	120.35 (11)
C2A—C1A—H1A	110.3	C11A—C10A—C15A	119.31 (12)
C9A—N1A—C1A	117.08 (11)	C9A—C10A—C15A	120.30 (11)
C9A—N1A—H1N1	115.0 (12)	C18B—C9B—N1B	122.14 (15)
C1A—N1A—H1N1	112.7 (12)	C18B—C9B—C10B	120.75 (14)
C3A—O1A—H1OA	106.1 (14)	N1B—C9B—C10B	117.02 (13)
C7A—C2A—C3A	118.36 (13)	C12A—C11A—N2A	121.94 (12)
C7A—C2A—C1A	120.67 (13)	C12A—C11A—C10A	120.34 (12)
C3A—C2A—C1A	120.97 (12)	N2A—C11A—C10A	117.56 (12)
C11A—N2A—C1A	117.26 (10)	C9B—C10B—C11B	120.84 (12)
C11A—N2A—H1NA	112.9 (12)	C9B—C10B—C15B	119.55 (15)
C1A—N2A—H1NA	111.1 (12)	C11B—C10B—C15B	119.53 (15)
C3B—O1B—H1OB	107.7 (17)	C11A—C12A—C13A	119.88 (14)
C9B—N1B—C1B	114.90 (11)	C11A—C12A—H12A	120.1
C9B—N1B—H1NB	113.0 (10)	C13A—C12A—H12A	120.1
C1B—N1B—H1NB	112.5 (10)	C12B—C11B—N2B	122.37 (15)
N2B—C1B—N1B	106.09 (10)	C12B—C11B—C10B	120.51 (14)
N2B—C1B—C2B	110.09 (11)	N2B—C11B—C10B	117.02 (12)
N1B—C1B—C2B	110.99 (10)	C14A—C13A—C12A	121.31 (14)
N2B—C1B—H1B	109.9	C14A—C13A—H13A	119.3
N1B—C1B—H1B	109.9	C12A—C13A—H13A	119.3
C2B—C1B—H1B	109.9	C11B—C12B—C13B	119.89 (19)
O1A—C3A—C4A	117.37 (13)	C11B—C12B—H12B	120.1
O1A—C3A—C2A	122.12 (12)	C13B—C12B—H12B	120.1
C4A—C3A—C2A	120.50 (14)	C13A—C14A—C15A	120.62 (13)
C7B—C2B—C3B	118.45 (12)	C13A—C14A—H14A	119.7
C7B—C2B—C1B	120.52 (11)	C15A—C14A—H14A	119.7
C3B—C2B—C1B	121.02 (11)	C14B—C13B—C12B	121.03 (18)
C11B—N2B—C1B	116.45 (11)	C14B—C13B—H13B	119.5
C11B—N2B—H2NB	114.1 (10)	C12B—C13B—H13B	119.5
C1B—N2B—H2NB	114.4 (10)	C14A—C15A—C16A	123.37 (13)
C5A—C4A—C3A	119.71 (16)	C14A—C15A—C10A	118.52 (12)
C5A—C4A—H4A	120.1	C16A—C15A—C10A	118.08 (13)
C3A—C4A—H4A	120.1	C13B—C14B—C15B	121.50 (16)
O1B—C3B—C4B	117.87 (12)	C13B—C14B—H14B	119.3
O1B—C3B—C2B	121.81 (12)	C15B—C14B—H14B	119.3
C4B—C3B—C2B	120.30 (12)	C17A—C16A—C15A	120.50 (13)
C6A—C5A—C4A	120.55 (14)	C17A—C16A—H16A	119.8
C6A—C5A—H5A	119.7	C15A—C16A—H16A	119.8
C4A—C5A—H5A	119.7	C16B—C15B—C14B	124.57 (18)
C5B—C4B—C3B	120.04 (14)	C16B—C15B—C10B	117.87 (18)
C5B—C4B—H4B	120.0	C14B—C15B—C10B	117.52 (18)
C3B—C4B—H4B	120.0	C16A—C17A—C18A	121.78 (13)
C5A—C6A—C7A	119.76 (16)	C16A—C17A—H17A	119.1
C5A—C6A—H6A	120.1	C18A—C17A—H17A	119.1
C7A—C6A—H6A	120.1	C17B—C16B—C15B	121.10 (17)
C4B—C5B—C6B	120.27 (13)	C17B—C16B—H16B	119.4
C4B—C5B—H5B	119.9	C15B—C16B—H16B	119.4
C6B—C5B—H5B	119.9	C9A—C18A—C17A	119.91 (14)
C6A—C7A—C2A	121.10 (15)	C9A—C18A—H18A	120.0
C6A—C7A—H7A	119.4	C17A—C18A—H18A	120.0
C2A—C7A—H7A	119.4	C9B—C18B—C17B	118.95 (19)
C5B—C6B—C7B	119.85 (14)	C9B—C18B—H18B	120.5
C5B—C6B—H6B	120.1	C17B—C18B—H18B	120.5
C7B—C6B—H6B	120.1	C16B—C17B—C18B	121.76 (19)
C18A—C9A—N1A	123.03 (13)	C16B—C17B—H17B	119.1
C18A—C9A—C10A	119.42 (12)	C18B—C17B—H17B	119.1
			
N2A—C1A—N1A—C9A	53.40 (15)	C1A—N2A—C11A—C10A	27.40 (17)
C2A—C1A—N1A—C9A	171.78 (12)	C9A—C10A—C11A—C12A	−177.51 (12)
N1A—C1A—C2A—C7A	110.84 (15)	C15A—C10A—C11A—C12A	0.02 (18)
N2A—C1A—C2A—C7A	−132.41 (14)	C9A—C10A—C11A—N2A	−1.84 (18)
N1A—C1A—C2A—C3A	−68.59 (16)	C15A—C10A—C11A—N2A	175.69 (11)
N2A—C1A—C2A—C3A	48.16 (16)	C18B—C9B—C10B—C11B	177.89 (14)
N1A—C1A—N2A—C11A	−50.96 (15)	N1B—C9B—C10B—C11B	1.32 (18)
C2A—C1A—N2A—C11A	−169.89 (11)	C18B—C9B—C10B—C15B	1.2 (2)
C9B—N1B—C1B—N2B	57.04 (13)	N1B—C9B—C10B—C15B	−175.39 (12)
C9B—N1B—C1B—C2B	176.61 (11)	N2A—C11A—C12A—C13A	−174.43 (13)
C7A—C2A—C3A—O1A	−177.96 (13)	C10A—C11A—C12A—C13A	1.0 (2)
C1A—C2A—C3A—O1A	1.5 (2)	C1B—N2B—C11B—C12B	−155.68 (12)
C7A—C2A—C3A—C4A	1.3 (2)	C1B—N2B—C11B—C10B	27.86 (16)
C1A—C2A—C3A—C4A	−179.23 (13)	C9B—C10B—C11B—C12B	−175.50 (12)
N2B—C1B—C2B—C7B	−118.22 (14)	C15B—C10B—C11B—C12B	1.21 (19)
N1B—C1B—C2B—C7B	124.63 (13)	C9B—C10B—C11B—N2B	1.03 (18)
N2B—C1B—C2B—C3B	61.14 (16)	C15B—C10B—C11B—N2B	177.74 (12)
N1B—C1B—C2B—C3B	−56.01 (16)	C11A—C12A—C13A—C14A	−0.9 (2)
N1B—C1B—N2B—C11B	−55.21 (14)	N2B—C11B—C12B—C13B	−177.43 (13)
C2B—C1B—N2B—C11B	−175.37 (11)	C10B—C11B—C12B—C13B	−1.1 (2)
O1A—C3A—C4A—C5A	178.05 (14)	C12A—C13A—C14A—C15A	−0.3 (2)
C2A—C3A—C4A—C5A	−1.3 (2)	C11B—C12B—C13B—C14B	−0.3 (3)
C7B—C2B—C3B—O1B	−176.49 (14)	C13A—C14A—C15A—C16A	179.26 (14)
C1B—C2B—C3B—O1B	4.1 (2)	C13A—C14A—C15A—C10A	1.3 (2)
C7B—C2B—C3B—C4B	2.2 (2)	C11A—C10A—C15A—C14A	−1.20 (18)
C1B—C2B—C3B—C4B	−177.14 (13)	C9A—C10A—C15A—C14A	176.34 (12)
C3A—C4A—C5A—C6A	0.3 (3)	C11A—C10A—C15A—C16A	−179.24 (11)
O1B—C3B—C4B—C5B	176.17 (15)	C9A—C10A—C15A—C16A	−1.70 (18)
C2B—C3B—C4B—C5B	−2.6 (2)	C12B—C13B—C14B—C15B	1.5 (3)
C4A—C5A—C6A—C7A	0.5 (3)	C14A—C15A—C16A—C17A	−176.90 (14)
C3B—C4B—C5B—C6B	0.8 (2)	C10A—C15A—C16A—C17A	1.0 (2)
C5A—C6A—C7A—C2A	−0.4 (3)	C13B—C14B—C15B—C16B	176.13 (19)
C3A—C2A—C7A—C6A	−0.5 (2)	C13B—C14B—C15B—C10B	−1.3 (3)
C1A—C2A—C7A—C6A	−179.92 (14)	C9B—C10B—C15B—C16B	−0.9 (2)
C4B—C5B—C6B—C7B	1.4 (3)	C11B—C10B—C15B—C16B	−177.64 (15)
C1A—N1A—C9A—C18A	153.25 (13)	C9B—C10B—C15B—C14B	176.72 (14)
C1A—N1A—C9A—C10A	−31.88 (17)	C11B—C10B—C15B—C14B	0.0 (2)
C5B—C6B—C7B—C2B	−1.7 (2)	C15A—C16A—C17A—C18A	−0.2 (2)
C3B—C2B—C7B—C6B	−0.1 (2)	C14B—C15B—C16B—C17B	−176.93 (19)
C1B—C2B—C7B—C6B	179.29 (14)	C10B—C15B—C16B—C17B	0.5 (3)
C18A—C9A—C10A—C11A	179.05 (12)	N1A—C9A—C18A—C17A	174.10 (13)
N1A—C9A—C10A—C11A	3.99 (17)	C10A—C9A—C18A—C17A	−0.7 (2)
C18A—C9A—C10A—C15A	1.53 (19)	C16A—C17A—C18A—C9A	0.0 (2)
N1A—C9A—C10A—C15A	−173.53 (11)	N1B—C9B—C18B—C17B	175.37 (15)
C1B—N1B—C9B—C18B	151.26 (14)	C10B—C9B—C18B—C17B	−1.0 (2)
C1B—N1B—C9B—C10B	−32.21 (16)	C15B—C16B—C17B—C18B	−0.4 (3)
C1A—N2A—C11A—C12A	−157.00 (13)	C9B—C18B—C17B—C16B	0.6 (3)

### Hydrogen-bond geometry (Å, º)

**Table:**

*D*—H···*A*	*D*—H	H···*A*	*D*···*A*	*D*—H···*A*
O1*A*—H1*OA*···N1*A*	0.86 (2)	2.66 (2)	3.1072 (16)	113.8 (17)
O1*A*—H1*OA*···N2*A*	0.86 (2)	2.03 (2)	2.7763 (16)	144.6 (19)
O1*B*—H1*OB*···N1*B*	0.84 (2)	2.20 (3)	2.8835 (16)	138 (2)
O1*B*—H1*OB*···N2*B*	0.84 (2)	2.47 (2)	3.0196 (16)	123 (2)
N1*B*—H1*NB*···O1*A*	0.865 (17)	2.331 (17)	3.1608 (18)	160.8 (14)
